# Supplementary material for: Two roles for the yeast transcription coactivator SAGA and a set of genes redundantly regulated by TFIID and SAGA
Source: eLife. 2020 Jan 8;9:e50109. doi: 10.7554/eLife.50109 (PMC6977968; doi:10.7554/eLife.50109)
Supplement: Supplementary file 9. — Strains were validated using a combination of genetic assays, phenotypic analysis, Western analysis, PCR analysis and nucleic acid sequencing. [file elife-50109-supp9.docx]

**Supplementary File 9:** Yeast Strains

| **Strain** | **Description** | **Genotype** | **Reference** |
| --- | --- | --- | --- |
| BY4705 | Wild type (WT) | *matα Δade2::hisG his3Δ200 leu2Δ0 lys2Δ0 met15Δ0 trp1Δ63 ura3Δ0* | (Brachmann et al., 1998) |
| SHY565 | *RPB3*-Flag | *matα Δade2::hisG his3Δ200 leu2Δ0 lys2Δ0 met15Δ0 trp1Δ63 ura3Δ0 RPB3* - Flag3::KanMX | (Warfield et al., 2017) |
| SHY1187 | *RPB3*-Flag, *spt3Δ* | *matα Δade2::hisG his3Δ200 leu2Δ0 lys2Δ0 met15Δ0 trp1Δ63 ura3Δ0 RPB3* - Flag3::KanMX *spt3Δ*::NAT MX | This work |
| SHY851 | *RPB3*-Flag, *spt7Δ* | *matα Δade2::hisG his3Δ200 leu2Δ0 lys2Δ0 met15Δ0 trp1Δ63 ura3Δ0* spt7 delta::HPH | This work |
| SHY897 | *spt20Δ* | *matα Δade2::hisG his3Δ200 leu2Δ0 lys2Δ0 met15Δ0 trp1Δ63 ura3Δ0* spt20*Δ*::KanMX | This work. |
| SHY1320 | *ubp8Δ* | *matα Δade2::hisG his3Δ200 leu2Δ0 lys2Δ0 met15Δ0 trp1Δ63 ura3Δ0* ubp8 delta::KanMX | This work |
| YKL60 | *gcn5Δ* | *mat a his3Δ1 leu2Δ0 met15Δ0 ura3Δ0 gcn5Δ*::KanMX | (Lee et al., 2005) From Workman lab |
| SHY1175 | *RPB3*-Flag, *SPT3+SPT20*-degron | *matα Δade2::hisG his3Δ200 leu2Δ0 lys2Δ0 met15Δ0 trp1Δ63 ura3Δ0 RPB3*-3x Flag::NAT MX *pGPD1-OSTIR::HIS3 SPT20*-3xV5-mini degron IAA7::KanMX *SPT3*-3xV5-IAA7degron::*URA3* | This work |
| SHY1176 | *RPB3-*Flag, *SPT3+SPT7*-degron | *matα Δade2::hisG his3Δ200 leu2Δ0 lys2Δ0 met15Δ0 trp1Δ63 ura3Δ0 RPB3*-3x Flag::NAT MX *pGPD1-OSTIR::HIS3*  *SPT7*-3xV5-IAA7 degron::KanMX *SPT3*-3xV5 IAA7degron:: *URA3* | This work |
| SHY1043 | *RPB3-*Flag, *TAF13*-degron | *matα Δade2::hisG his3Δ200 leu2Δ0 lys2Δ0 met15Δ0 trp1Δ63 ura3Δ0 RPB3*-3x Flag::NAT MX *pGPD1-OSTIR::HIS3*  *TAF13*-3xV5 IAA7- degron::KanMX | This work |
| SHY1039 | *RPB3-*Flag, *TAF1*-degron | *mat alpha delta ade2::hisG his3 delta 200 leu2 delta 0 lys2 delta 0 met15 delta 0 trp1 delta 63 ura3 delta 0* *RPB3*-3x Flag::NAT MX *pGPD1-OSTIR::HIS3*  *TAF1*-3xV5- IAA7 degron::KanMX | This work |
| SHY1041 | *RPB3-*Flag, *TAF7*-degron | *matα Δade2::hisG his3Δ200 leu2Δ0 lys2Δ0 met15Δ0 trp1Δ63 ura3Δ0 RPB3*-3x Flag::NAT MX *pGPD1-OSTIR::HIS3*  *TAF7*-3xV5- IAA7 degron::KanMX | This work |
| SHY1291 | *TAF13+SPT7*-degron | *matα Δade2::hisG his3Δ200 leu2Δ0 lys2Δ0 met15Δ0 trp1Δ63 ura3Δ0 RPB3*-3x Flag::NAT MX *pGPD1-OSTIR::HIS3*  *TAF13*-3xV5- IAA7 degron::KanMX  *SPT7*-3xV5 IAA7-degron::*URA3* | This work |
| SHY1292 | *TAF13+SPT3*-degron | *matα Δade2::hisG his3Δ200 leu2Δ0 lys2Δ0 met15Δ0 trp1Δ63 ura3Δ0 RPB3*-3x Flag::NAT MX *pGPD1-OSTIR::HIS3*  *TAF13*-3xV5- IAA7 degron::KanMX  *SPT3* -3xV5 IAA7-degron::*URA3* | This work |
| SHY1228 | *TAF13*-MNase | *matα Δade2::hisG his3Δ200 leu2Δ0 lys2Δ0 met15Δ0 trp1Δ63 ura3Δ0 TAF13-*MNase::*TRP1* | This work |
| RDY55 | *TAF1*-MNase | *matα Δade2::hisG his3Δ200 leu2Δ0 lys2Δ0 met15Δ0 trp1Δ63 ura3Δ0 TAF1-*MNase*::TRP1* | This work |
| SHY1227 | *TAF7*-MNase | *matα Δade2::hisG his3Δ200 leu2Δ0 lys2Δ0 met15Δ0 trp1Δ63 ura3Δ0 TAF7-*MNase::*TRP1* | This work |
| SHY1185 | *SPT7*-MNase | *matα Δade2::hisG his3Δ200 leu2Δ0 lys2Δ0 met15Δ0 trp1Δ63 ura3Δ0 SPT7-*MNase::*TRP1* | This work |
| SHY1184 | *SPT3*-MNase | *matα Δade2::hisG his3Δ200 leu2Δ0 lys2Δ0 met15Δ0 trp1Δ63 ura3Δ0 SPT3-MNase::TRP1* | This work |
| SHY1058 | *S. pombe* WT / RPB3-Flag | *S. pombe* mating type h-  *RPB3-*3xFlag::KanMX | From G. Smith lab-FredHutch |

**References:**

Brachmann C, Davies A, Cost G, Caputo E, Li J, Hieter P, Boeke J. 1998. Designer deletion strains derived from Saccharomyces cerevisiae S288C: a useful set of strains and plasmids for PCR-mediated gene disruption and other applications. *Yeast (Chichester, England)* **14**:115–132. doi:10.1002/(sici)1097-0061(19980130)14:2<115::aid-yea204>3.0.co;2-2

Lee K, Florens L, Swanson S, Washburn M, Workman J. 2005. The Deubiquitylation Activity of Ubp8 Is Dependent upon Sgf11 and Its Association with the SAGA Complex. *Mol Cell Biol* **25**:1173–1182. doi:10.1128/mcb.25.3.1173-1182.2005

Warfield L, Srinivas Ramachandran, Baptista T, Devys D, Tora L, Hahn S. 2017. Transcription of Nearly All Yeast RNA Polymerase II-Transcribed Genes Is Dependent on Transcription Factor TFIID. *Molecular cell* **68**:118-129.e5. doi:10.1016/j.molcel.2017.08.014
